# Evolving data access policy: The Canadian context

**DOI:** 10.1139/facets-2016-0002

**Published:** 2016-09-15

**Authors:** Stephanie O.M. Dyke, Katie M. Saulnier, Tomi Pastinen, Guillaume Bourque, Yann Joly

**Affiliations:** a Department of Human Genetics, Centre of Genomics and Policy, Faculty of Medicine, McGill University, Montreal, QC H3A 0G1, Canada; b Department of Human Genetics, Epigenomics Mapping Centre, Faculty of Medicine, McGill University, Montreal, QC H3A 0G1, Canada

**Keywords:** data sharing, data access, ethics, privacy, consent, controlled access

## Abstract

In setting up a data access policy to share controlled access data from the McGill Epigenomics Mapping Centre (EMC), an International Human Epigenome Consortium (IHEC) partner project, we encountered ethical and legal challenges that are likely to be relevant to other researchers sharing data, especially from Canadian projects. We discuss our solutions to the following data-sharing challenges, based on comparative legal and policy analysis: (1) providing access to data to a growing number of researchers; (2) maintaining Canadian privacy standards while sharing controlled access data internationally; (3) freedom of information requests; and (4) providing more incentives for researchers to share pre-publication data.

## Introduction

The rise of large-scale data and data sharing has been accompanied by new ethical and legal challenges regarding the stewardship of large amounts of personal information. Although open access platforms, in principle, release data to facilitate broad data use, they may leave sensitive research data vulnerable to unethical behavior and confidentiality breaches. “Controlled access” data access mechanisms provide a way for researchers to utilize data while protecting it through data access agreements and policies that strike a balance between accessibility and control remaining in the hands of data stewards (Toronto International Data Release Workshop 2009; Ramos et al. 2013; Milius et al. 2014). Controlled access is recommended when the risk that data could potentially be misused is likely high. We recently conducted an assessment of the risks posed by sharing epigenome methylation data, one of the main data types produced by epigenome mapping studies (Dyke et al. 2015). This study led us to suggest masking those sites showing the most genetic contribution in open access data (at common single nucleotide polymorphism (SNP) positions or at the individual’s SNPs) and sharing some rare disease and ethnicity data through controlled access. Typically, controlled access is used by large-scale genomic and epigenomic projects to share composite genomic and clinical data that are associated with a unique, but not directly identified, person. Researchers applying for access to controlled data are approved based on their qualifications and the nature of their research proposal. “Approved” researchers must also agree to specific conditions to obtain authorization to use controlled access data, such as keeping the data secure, not sharing it with unauthorized third parties and only using it for approved purposes to protect the interests of research participants. These conditions are stipulated in a “Data Access Agreement” (DAA; Joly et al. 2011).

The McGill Epigenomics Mapping Centre (EMC) and Data Coordination Centre, established in 2012 in Montreal, Quebec, Canada, were designed to support “large-scale human epigenome mapping for a broad spectrum of cell types and diseases” (McGill Epigenomics Mapping Centre 2015). Data generated at the EMC include transcriptome (RNA-seq and small molecule RNA sequencing (smRNA-seq)), genome-wide methylome (whole genome bisulfite sequencing (WGBS)), targeted methylome (methylC-capture sequencing (MCC-seq)), histone modification (ChIP-seq), and accessible/active chromatin (ATAC-seq). As an International Human Epigenome Consortium (IHEC) partner project, one goal of the EMC is to “share its data with the entire research community to accelerate the translation of new knowledge of health and diseases” (McGill Epigenomics Mapping Centre 2014). The EMC’s research goals are, therefore, facilitated by the use of a DAA that provides sufficient protection for research participants’ data while ensuring that it is made available in a way that is straightforward and allows access to the greatest number of qualified researchers.

The EMC data access policy was developed with a number of innovative provisions intended to enhance the safe and straightforward transfer of data to qualified researchers, and to address the Canadian legal context. We adopted features of DAAs designed to help facilitate understanding and compliance by researchers who may not be familiar with the language of standard legal contracts, including brevity, the use of clear and simple language, and limiting the contract’s content to essential elements, to “reduce time for negotiation between the study administrators and researchers” (Knoppers et al. 2013). We also drew on our experience with the International Cancer Genome Consortium (ICGC) Data Access Compliance Office (Milius et al. 2014).^1^

We discuss provisions of the EMC DAA here as we hope they may be of use to other institutions aiming to keep data protected while still encouraging innovative research. Furthermore, concerns over the lack of consistency in data access principles and agreements have been reported, even within programs, and these may be addressed through greater access to and explanation of existing policies and DAAs (Lowrance 2006). The EMC DAA is available on the EMC Data Access Policy web page.

## A broad range of data users

There are good reasons for taking into consideration the scientific qualifications of individuals applying to use controlled data in their research. However, the required level of competence is not obvious, and how the qualifications of applicants are reviewed and approved is often not well defined in data access procedures (Shabani et al. 2015). A rather standard approach has been to request evidence of relevant scientific publications in academic journals (see Table 1). However, strict adherence to a “three relevant publications” guideline, for example, may exclude competent researchers who have not had the opportunity to publish very much, including upcoming researchers with innovative ideas and researchers in industry or in fields that do not prioritize publication in scientific journals. We, therefore, sought to explicitly provide flexibility as to the evidence of qualification required of the main applicant and included the following in the EMC access application form, as we do not wish to discourage researchers from applying, though they may need the sponsorship of a more senior researcher:

If you have not authored or co-authored three relevant publications please describe your relevant expertise or experience in no more than 300 words.

It is important to note that if they are based at a university or research institute, the main applicants for access to EMC data should be group leaders, and are, therefore, highly qualified, though they may then list members of their teams as data users. Industry researchers should have an equivalent position within their company (e.g., scientific director, senior researcher).

In our view, this flexibility can readily be managed by data access committees (DACs) that include domain experts to evaluate such descriptions of expertise and experience alongside research proposals. The EMC DAC includes four members with diverse scientific training.

While this should encourage many qualified researchers to access the data, it does not extend access to journalists or interested members of the public, which has been done in Europe for access to summary clinical trials data for example (European Medicines Agency 2014). Another important group of eventual, potential data users are clinical care professionals. We envisage that unnecessary barriers to data access will continue to fall as public demand mounts and technological developments further facilitate privacy protection.

## Canadian privacy standards

Canadian privacy standards as they apply to health research stem from both legislation and policy documents. Additionally, from the legal perspective, the mix of federal and provincial jurisdictions in Canadian law alongside the separation of the public and private sectors in regulations means that understanding the Canadian privacy framework is complicated in the best of circumstances. Clauses in DAAs that cater to international researchers are particularly challenging, as national laws are only now beginning to catch up with reality on the question of trans-border data flow. Reminding researchers of obligations and requirements external to those listed in the DAA has been recommended by experts in the field of health research (Lowrance 2006). As referring to national law may not be very useful when data from different jurisdictions are brought together and shared under one project (e.g., ICGC), referring to international policy instruments can then be helpful (see Table 1). The EMC shares data generated in the Canadian context. We, therefore, included in our access agreement references to the *Tri-Council Policy Statement: Ethical Conduct for Research Involving Humans* (CIHR et al. 2014) and to the main provincial privacy legislation in Quebec for the public and private sectors (Quebec 1994, 2010), along with references to international policy statements of the IHEC (2013), the genomics research community (Wellcome Trust 2003; Toronto International Data Release Workshop 2009), and the Organization for Economic Cooperation and Development (OECD 2006), and to an NIH policy as it provided the most suitable guidance on licensing genomic inventions (National Institutes of Health 2005):

The User and User Institution(s) understand and acknowledge that EMC Data is protected by and subject to applicable laws and ethical guidelines, which may include without limitation the *Tri-Council Policy Statement* (TCPS2), Quebec’s *An Act Respecting Access to Documents Held by Public Bodies and the Protection of Personal Information*, and Quebec’s *An Act Respecting Protection of Personal Information in the Private Sector*.

Canadian ethical standards for the privacy of research participants are founded on three core principles: respect for persons, concern for welfare, and justice; standards that are derived from the Canadian Charter of Rights and Freedoms (Constitution Act 1982; CIHR et al. 2014). These standards concern protection from potential harms as well as allowing participants to make informed decisions about the use of their information and biological material (CIHR et al. 2014). The TCPS2 officially applies only to projects funded by one of Canada’s three federal research funding agencies, but its principles draw on general Canadian norms, and it places the responsibility for upholding the ethical duty of privacy protection in the hands of researchers (CIHR et al. 2014). Institutions wishing to receive and administer research funds from Canada’s three federal research agencies must agree to comply with the standards set out in the TCPS2. Highlighting the significance of ethical guidelines such as the TCPS2 in our DAA helps to ensure that data being accessed and handled in foreign jurisdictions are subject to these same standards. Although we recognize that many users will not opt to take the time to fully read through these guidelines and are not equipped to fully understand laws, it is nevertheless important to give researchers and their organizations a frame of reference for the ethical context in which they will be working while accessing EMC datasets. This is also helpful in case of dispute.

The EMC also requests from applicants an attestation of (or documentation proving) compliance with local ethical requirements (e.g., Research Ethics Committee approval for the research to be conducted using the data) if required in the applicants’ jurisdiction.

The Canadian legal framework that protects individuals’ right to privacy and the right to determine the use of their personal information encompasses laws at both the federal and provincial levels. Each province or territory has legislation on the protection of personal information, and some jurisdictions have specific legislation addressing personal health information; in Quebec, the protection of health data is subsumed under the broader private sector legislation. Conflicts between provincial and federal laws end with the federal law superseding the provincial, except in cases where the provincial law has been deemed “substantially similar” to the federal law (Office of the Privacy Commissioner of Canada 2013), in this case, namely, *The Personal Information Protection and Electronic Documents Act* (PIPEDA) in the private sector, and the *Privacy Act* for the public sector (Government of Canada 1985, 2000; Saulnier and Joly 2016).

Additionally, users are reminded in the DAA not to send data outside of the institution to which controlled access has been granted and they are requested to report demands for disclosure:

The User and User Institution(s) agree that except as expressly provided in section F “Research Project”, or with the express authorization of EMC, no identifiable data may be sent outside the User Institution(s) by the User and User Institution(s) for any reason. The User and User Institution(s) agree to immediately report to EMC any demand for disclosure.

The intention behind the inclusion of this paragraph is twofold: to ensure that the user understands the primacy of their agreement with the EMC when faced with possibly competing interests and to ensure that the EMC is made aware of situations where an outside entity not party to the DAA is seeking access to confidential information. This approach is especially important in Canada, as PIPEDA regulates international trans-border data flow using an accountability model that is considered to be implicit in Quebec’s own parallel legislation; the organization that controls the data is responsible for ensuring that Canadian privacy standards continue to be met wherever the data goes (Office of the Privacy Commissioner of Canada 2004, 2012). This type of clause has also been adopted by the BC Cancer Agency (see Table 1).

Finally, regarding the question of trans-border data flow, the international nature of scientific research can make it difficult for researchers to ascertain under which jurisdiction disputes will fall and such disputes may be prohibitively expensive abroad. We, therefore, included a clause regarding the governing law to ensure that potential disputes would be held in Quebec as this is the Canadian province where the EMC is located. Our decision to include a jurisdiction for settling disputes is not without precedent even within data access agreements; of the other agreements we examined in creating our own, the BC Cancer Agency also included such a jurisdictional provision (see Table 1). Moreover, the inclusion of governing law or forum selection clauses is a practice that promotes legal previsibility and is common in contract law when parties will be operating in different jurisdictions (Brown 2012).

## Data protection law and freedom of information

One of the results of the complicated legal framework for privacy law is that it becomes very difficult for a layperson to be sure of their rights and responsibilities under laws that are distinct from the explicit provisions of the DAA. Complex legal situations may arise whereby data custodians and data users may be unclear as to their obligations in law. A particular example of this is the sometimes conflicting demands of data protection law and freedom of information (FOI) law. FOI requests can create conflict not only with regard to the contractual agreements between data stewards and accessing researchers and institutions, but also between these bodies and the original patients or research participants who may have consented to share their information under the explicit agreement that it would remain private. Although FOI law has proven essential in holding institutions, including their researchers, to account, if sharing EMC data publicly were ethical and feasible then we would not be using a controlled access mechanism. Quebec’s disclosure exceptions outline situations under which a public body is permitted to release personal information without consent of the subject of that information (Quebec 2010). The article is phrased in the conditional; it refers to situations in which a public body *may* release personal information without the consent of parties involved, not situations where it is required to do so. On balance and in light of the possible conflicts, we, therefore, adopted in our DAA a reference to the binding nature of this agreement even in the face of discretionary disclosure exceptions of Quebec’s (2010) *An Act Respecting Access to Documents Held by Public Bodies and the Protection of Personal Information*, stating that:

EMC Data must only be used and disclosed as expressly provided in this agreement, even in the case of discretionary freedom of information disclosure exceptions outlined in Quebec’s *An Act Respecting Access to Documents Held by Public Bodies and the Protection of Personal Information* or documents of similar force and effect.

FOI requests are not typically referred to in DAAs (see Table 1). By explicitly stating that EMC data cannot be disclosed even under Quebec’s (2010) *An Act Respecting Access to Documents Held by Public Bodies and the Protection of Personal Information* FOI provisions, we wish to clarify our expectations regarding the rights and responsibilities of the parties under Quebec’s broader privacy framework.

## Data-sharing incentives

As policies requiring data sharing in genomics research have become commonplace, researchers may still have concerns about losing credit for their work through data sharing (Dyke and Hubbard 2011). A study of data sharing and withholding in academic genetics conducted over a decade ago showed the effort and cost of sharing, and protecting the ability to publish, were regularly given as reasons for not sharing (Campbell et al. 2002). Similar concerns have been identified more recently (Tenopir et al. 2011, 2015). Initiatives such as the Bioresource Impact Factor (BRIF) and CoBRA Citation of BioResources in journal articles guidelines aim to develop comprehensive systems for attribution of reward for sharing research resources through standardized citation and micro-citation of bioresources (Cambon-Thomsen et al. 2011; Bravo et al. 2015). We, therefore, clarified how to acknowledge the EMC’s contribution to projects using shared data in the DAA:

The User and User Institution(s) will acknowledge the source of the EMC Data such as follows in the methods section of the manuscript if possible or elsewhere in the main text of the manuscript: “This research used data shared by the McGill Epigenomics Mapping Centre and it is available from the European Genome-phenome Archive of the European Bioinformatics Institute (accession numbers: study EGAS00001000995 and dataset (s) EGAD00 …)”. Please also cite: McGill Epigenomics Mapping Centre (2015). Dataset from EGA Study EGAS00001000995 (Data file). Available from http://epigenomesportal.ca/edcc.

As journal publications and their citation remain the predominant form in which funding agencies and institutions are likely to pass on reward to investigators sharing data, including relevant publications for data users to cite in acknowledgement instructions should be encouraged, either those pertaining to the dataset or to the data resource or research project.

## Conclusion

The details of funders’ and research projects’ data-sharing policies and the DAAs that govern access to data have an important impact on the research environment, from determining how widely data will be shared, to upholding ethical standards of data use in research, to fostering greater acknowledgment of scientists sharing data. Although some trends, such as expanding the number of researchers who are able to access and analyze data, are clearly seen internationally, we believe international collaborations and data-sharing initiatives will also benefit from further understanding of local norms and standards, and legal requirements in particular. We also believe that coherence between national and international privacy frameworks could be improved. Canada is an active partner in numerous international scientific projects and we hope the data access provisions we describe here may serve to reinforce its participation in science around the world.

## Figures and Tables

**Table 1:** Comparative analysis of DAAs from epigenomic (BC Cancer Agency (BCCA), Blueprint, German Epigenome Program (DEEP), EMC) and genomic (ICGC) data sharing projects.

	BCCA	Blueprint	DEEP	ICGC	EMC
Evidence of competence requested	Describe full experience and expertise. A publication list MUST be provided for the applicant, co-applicants, and Ph.D. supervisors where Ph.D. students have applied.	Describe full experience and expertise. A publication list MUST be provided for the applicant, co-applicants, and Ph.D. supervisors where Ph.D. students have applied.	Describe full experience and expertise. A publication list MUST be provided for the applicant, co-applicants, and Ph.D. supervisors where Ph.D. students have applied.	Main applicant MUST be a PI and list three publications.	Main applicant should be a PI and list three publications or describe relevant experience/expertise.
Laws specifically referred to	Provincial and federal freedom of information and privacy laws	None, but it includes a statement about user’s responsibility for complying with applicable laws.	None	None	Provincial freedom of information and privacy laws
Policies referred to	None	Fort Lauderdale Guidelines	IHEC guidelines	Fort Lauderdale Guidelines, Toronto Statement, NIH Best Practices for Licensing of Genomic Inventions, OECD Guidelines for the Licensing of Genetic Inventions, and ICGC policies.	Fort Lauderdale Guidelines, Toronto Statement, NIH Best Practices for Licensing of Genomic Inventions, OECD Guidelines for the Licensing of Genetic Inventions, TCPS2, and IHEC policies.
Is the jurisdiction for disputes set?	Yes	No	No	No	Yes
Does the DAA state what to do if faced with demands for access?	Yes. Immediately report to BCCA any foreign demand for disclosure.	No	No	No	Yes. Immediately report to EMC any demand for disclosure.
Does the DAA provide guidance on FOI requests?	No	No	No	No	Yes. Data must only be used and disclosed as expressly provided in this agreement, even in the case of discretionary freedom of information disclosure exceptions outlined in Quebec’s (2010) *An Act Respecting Access to Documents Held by Public Bodies and the Protection of Personal Information* or documents of similar force and effect.
What guidance does the DAA give on acknowledgements?	The recipient agrees to acknowledge the contribution of the Study Investigator in any and all oral and written presentations, disclosures, and publications resulting from any and all analyses of data.	Authors who use data from the project must acknowledge Blueprint using the following wording: *This study makes use of data generated by the Blueprint Consortium. A full list of the investigators who contributed to the generation of the data is available from* www.blueprint-epigenome.eu. *Funding for the project was provided by the European Union’s Seventh Framework Programme (FP7/2007–2013) under grant agreement no. 282510 — BLUEPRINT. Users must also cite any relevant primary Blueprint publication (details of which can be found on the Blueprint website)*.	Must recognize the contribution of the Consortium and include a proper acknowledgement in any work based on whole or part on the DEEP data.	Must recognize the contribution of the consortium and include a proper acknowledgment in all reports or publications.	The source of the EMC data will be acknowledged such as follows in the methods sections of the manuscript if possible or elsewhere in the main text of the manuscript: *This research used data shared by the McGill Epigenomics Mapping Centre and it is available from the European Genome-phenome Archive of the European Bioinformatics Institute (accession numbers: study EGAS00001000995 and dataset(s) EGAD00 …)*. Please also cite: McGill Epigenomics Mapping Centre (2015). *Dataset from EGA Study EGAS00001000995 (Data file). Available from* http://epigenomesportal.ca/edcc.

## Footnotes

1 YJ is the ICGC Data Access Officer and a member of the ICGC Ethics and Policy Committee. SD is a former member of the ICGC Ethics and Policy Committee (2009–2013) and the ICGC Data Access Committee (2014–2015). GB is a member of the ICGC Data Coordination and Management Working Group.
